# Health Related Quality of Life Post Labour Induction with Misoprostol Versus Dinoprostone At Muhimbili National Hospital in Dar Es Salaam, Tanzania: A cross Sectional Study

**DOI:** 10.24248/eahrj.v4i1.622

**Published:** 2020-06-26

**Authors:** Jonas Kagwisage, Belinda S Balandya, Andrea B Pembe, Phares GM Mujinja

**Affiliations:** a Department of Obstetrics and Gynaecology, Muhimbili University of Health and Allied Sciences, Dar es Salaam, Tanzania; b Department of Behavioral Sciences, Muhimbili University of Health and Allied Sciences, Dar es Salaam, Tanzania

## Abstract

**Background:**

Labour induction using Misoprostol or Dinoprostone results to similar maternal and foetal clinical outcomes. However, the clinical outcome measures have rarely been combined with effects of interventions on patients' health related quality of life. This study aimed to assess postpartum health related quality of life of parturient after labour induction with vaginal administration of misoprostol versus dinoprostone.

**Methods:**

This was a comparative cross sectional study in which pregnant women who underwent labour induction with misoprostol and dinoprostone during the study period were included. Data were collected within 24 hours post-delivery using the 36 item short form health survey questionnaire which consists of 24 attributes distributed in five domains including bodily pains and physical performance three attributes each, mental health seven attributes, general health two attributes, social functioning six attributes and three attributes for labour induction satisfaction. We first estimated scores of all attributes in each domain using Likert scales and then the domain scores were converted into a 0 to 100 scales to express in percentage of total scores. Quality of life was compared in the two study groups using the independent samples T Test. Multivariate regression analysis was performed to control for marital status, gravidity, parity, baseline cervical status, time interval from induction to delivery and mode of delivery.

**Results:**

Women who received misoprostol reported better health related quality of life compared to those who received dinoprostone (mean score 92.89 vs. 87.25;*P*<.00). Misoprostol group had significantly higher scores in all domains of health related quality of life; reduced bodily pain (93.76 vs. 84.19;*P*<.00), physical performance (83.64 vs. 73.58;*P*<.00), mental health (96.40 vs. 93.55; *P*<.00), general health (93.78 vs. 90.23;*P*=.01), social functioning (94.81 vs. 91.25;*P*<.00) and satisfaction perceptions (94.96 vs. 90.71;*P*<.00).

**Conclusion:**

Health related quality of life information is of particular value in routine care of natal and postnatal mothers. Current and updated guidelines should address the impacts of labour induction interventions on maternal health related quality of life, and encourage the use of quality of life information in provision of holistic natal and postnatal care services. Clinical trials are recommended to determine the effectiveness of labour induction with either of the two methods and address the historical adverse outcomes associated to the use of misoprostol.

## BACKGROUND

Induction of labour is a commonly performed intervention in pregnancy worldwide^1–6^. It is offered when the benefits of ending the pregnancy outweigh the benefits of continuing with it^7,8^. Misoprostol and dinoprostone are the widely used labour induction methods in most countries.^9–12^ Misoprostol is a synthetic analogy of prostaglandin E1 whereas dinoprostone is a formulation of prostaglandin E2. In pregnancy, these prostaglandins acts on the cervix and uterus bringing about softening of the cervix^13^ and contraction of the uterine muscles^14^. Together, these effects cause effacement, and dilation of the cervix. Both misoprostol and dinoprostone are reported to be commonly used for cervical ripening and induction of labour in some of maternity units in Tanzania. At Muhimbili National Hospital (MNH), 52.5% of patients undergoing labour induction receive dinoprostone and 32.5% receive misoprostol^6^. While in Kilimanjaro Christian Medical Centre in northern Tanzania 18% of patients who undergo labour induction receive prostaglandins^18^. There is a wide range of literature on clinical outcomes of labour induction with misoprostol versus dinoprostone^16,17,19,20^. Labour induction using misoprostol results to more vaginal birth in some studies^16,17^ and less vaginal birth in other studies^20^. The incidences of abnormal uterine activity are similar in women receiving misoprostol and dinoprostone^17,20^ and more common among women receiving misoprostol in some studies^21,22^. Meconium staining of the liquor is reported commonly among women receiving misoprostol^17,19,20^ as well as in women receiving dinoprostone^16,22^. Incidences of low scores (Apgar score of <7 at five minutes) are similarly reported among women receiving misoprostol and dinoprostone^16^ and commonly reported among women receiving dinoprostone in other studies^17,20^.

Complication free vaginal delivery is the primary goal of labour induction^7,8^. A method that is associated with increased vaginal birth would enhance women's Health Related Quality of Life (HRQoL) post-delivery^23^. Postpartum women following vaginal birth are less likely to have problems with mobility, self-care, routine activities, pain or discomfort^23^. However, some studies reports no differences in postpartum HRQoL by mode of delivery^24^. Although misoprostol and dinoprostone appear to be equally effective, clinical outcome measures have rarely been combined with effects of interventions on patients' HRQoL.

Health related quality of life in the context of this study refers to a multi-dimensional concept of personal reported health status that comprises of domains related to physical, mental (eg, energy level, mood) and their correlates including health risks and conditions, functional status and social sustenance after receiving the treatment^25^. We conducted a quality of life study in order to identify HRQoL post-delivery with the aim of planning for appropriate nursing interventions to respond to health care needs of the involved patients and guide the choice of a better labour induction method. Therefore, the aim of the study was to assess and compare the postpartum HRQoL of women after induction of labour with vaginal administration of misoprostol versus dinoprostone in the form of tablets.

## METHODS

### Study Design and Settings

A comparative cross-sectional study was conducted at Muhimbili National Hospital, the tertiary referral and teaching hospital with capacity of 1650 beds located in Dar es Salaam, Tanzania. The hospital receives referral patients from Dar es Salaam regional hospitals and other hospitals from within the city and other regions in Tanzania. Pregnant women attend antenatal care run in clinics that are conducted from Monday to Friday by a team of Gynaecologists and Obstetricians, medical doctors, resident doctors and nurse midwife. On averages one hundred and fifty pregnant women attend per day.

Pregnant women at a gestation age of 28 weeks and above are admitted to the general obstetric unit. The unit has 8 wards located within two maternity blocks, with the capacity of 350 beds. The labour ward located within the main block has twenty delivery beds and a total of twenty five nurse midwives. The nurse midwives works in shifts of eight hours with each shift consisting of five nurses. About 60 deliveries are conducted at the labour ward every day of which nearly 5% are preceded by labour induction^6^.

Labour induction decision is made by the general obstetric unit team under the gynaecologist and obstetrician consultant during antenatal care clinic and ward rounds. The women scheduled for elective induction are admitted one day prior to the procedure for pre induction evaluation. Induction is initiated with either misoprostol or dinoprostone vaginal tablet. After the initiation of labour induction, women are observed in the wards until active phase of labour is established. Thereafter they are transferred to the labour ward for monitoring of labour progress, and possibly augmentation with intravenous oxytocin. Consequently, intermittent foetal heart rate monitoring is initiated in the labour ward using Doppler foetal monitor.

### Study Population

The study population consisted of two groups; a group of parturient undergoing induction of labour with misoprostol and those receiving dinoprostone.

### Sample Size

Sample Power analysis was used to estimate sample size of 200 study participants. The sample size was calculated using two proportions of vaginal deliveries following labour induction with misoprostol and dinoprostone^20^ by using the formula

$$\text{n}\left(\text{each group}\right)=\frac{{({\text{p}}_{0}{\text{q}}_{0}+{\text{p}}_{1}{\text{q}}_{1})\left({\text{z}}_{1-\text{a/2}}+{\text{z}}_{1-\text{b}}\right)}^{2}}{{\left({\text{p}}_{1}-{\text{p}}_{0}\right)}^{2}}$$

Where n = the minimum required sample size, z_(1-a/2)_= single sided confidence level which is 95% and Z_ß_ =power which was set at 80%, P_0_ =proportion of vaginal deliveries in the misoprostol group (78%) and P_1_ =proportion of vaginal deliveries in the dinoprostone group (59%). A sample of 90 participants for each group was initially estimated. Applying the adjusted sample size formula for anticipated 10% attrition rate, q=n/1-f (where q is adjusted sample size; n is original sample size; and, f is estimated non-response rate), the initial minimum sample size estimate was adjusted from 90 to 100 participants. Therefore, a minimum of 200 participants were required in this study.

### Sampling Procedure

We invited all pregnant women who were planned for labour induction during the antenatal care clinics and ward rounds, to participate in this study. Subsequent on obtaining written informed consent, all eligible expectant mothers were randomly selected until the target sample size was attained. The inclusion criteria were labour induction with either misoprostol or dinoprostone, gestation age of 28 weeks and above, a viable singleton pregnancy in cephalic presentation and intact membranes. We excluded women who had a known lethal foetal congenital anomaly, eclampsia and/or hypersensitivity to either of the products used for labour induction.

The protocol for pharmacological labour induction at MNH involves regular vaginal administration of a 25 microgram misoprostol tablet or a 3 milligram dinoprostone tablet. The gynaecologist and obstetrician consultant or resident medical doctor insert the tablet deep into the posterior fornix of the vagina at an interval of six hours. The number of doses depends on cervical status. The maximum was four for Misoprostol and two for dinoprostone. All study women were taken care of by the attending physician, and were managed according to the institution's protocol.

### Outcome Measures

Primary outcome measures were the quality of life domains assessed within 24 hours post-delivery. The measured quality of life domains were bodily pain, physical performance, mental health, general health, social functioning and labour induction satisfaction. Secondary outcome measures included the mode of delivery, and foetal-maternal complications which included foetal heart rate abnormalities, Apgar score of less than 7 at 5 minutes, meconium staining of the liquor, perineal trauma, postpartum haemorrhage, diarrhoea, vomiting and the need for blood transfusion.

### Data Collection

Data were collected from September 2017 to March 2018. A case record form was used to collect clinical data from patient file and delivery register. Information on maternal HRQol was obtained by using a Swahili version of the 36 item Short Form (SF-36) Health Survey generic questionnaire. The questionnaire consist of 24 attributes distributed in five domains including bodily pains and physical performance three attributes each, mental health seven attributes, general health two attributes, social functioning six attributes and three attributes for labour induction satisfaction. A team of investigators who are conversant in maternal health and fluent in both English and Swahili reviewed the translation individually and then in a group. Consensus was reached in all the items. Pretesting of the questionnaire was done among parturient undergoing induction of labour at a regional referral hospital in Dar es Salaam. The researchers participated to see whether the questions are clear and represent what is needed. The questionnaire was administered to study participants by the principle investigator during the first 24 hours following childbirth. The Likert scales (with five options) were used to form attributes in each domain. The item scores in each domain were scaled in a positive direction with the highest scores indicating better quality of life. Reliability testing of the generic version estimated using internal consistency method resulted in Cronbach's alpha scores of 0.94 for a sample of 3,445 patients^26^.

### Data Analysis

The collected information was entered into IBM SPSS Statistics for Windows version 23.0 (IBM Corp, Armonk, NY, USA). Data were analysed to compare the rates of different maternal and neonatal outcomes as well as quality of life in the two study groups using independent samples T test. The Multivariate regression analysis was performed to control for potential confounders including the marital status; gravidity, parity, baseline cervical status, time interval from induction to delivery and mode of delivery.

### Ethical Clearance

The study was ethically approved by the Muhimbili University of Health and Allied Sciences, Senate Research and Publication Committee with certificate reference numberMU/PGS/SAEC/ Vol.IX/. The permission to conduct this study was obtained from the MNH administration. Written consent was obtained from all women prior to the initiation of labour induction after explaining the aim and procedures of the study. Women were informed that they can withdrawal from study at any time and will continue receiving quality management of their labour according to the hospital protocols.

## RESULTS

A total of 228 women participated in the study, whereby 106 women were those induced with misoprostol and 122 with dinoprostone. (See Table 1)

**TABLE 1.** Women's Baseline Characteristics by Labour Induction Drug

Characteristics	Misoprostol (n = 106)	Dinoprostone (n = 122)	P value
**Age, years**			
Mean (SD)	28.60 (5.26)	30.10 (6.21)	0.06
**Gestation, weeks**			
Mean (SD)	38.16 (3.18)	38.37 (2.60)	0.59
**Parity, n (%)**			
0	32 (30.2)	51 (45.1)	0.13
1	31 (29.2)	35 (28.7)	0.13
2+	43 (40.6)	36 (29.5)	0.06
**Number of living children**			
Mean (SD)	1.32 (1.25)	1.06 (1.29)	0.12
**Marital status, n (%)**			
Married	84 (79.2)	103 (84.4)	0.46
Unmarried	18 (17.0)	17 (13.9)	0.47
Cohabiting	4 (3.8)	2 (1.6)	0.86
**Initial Bishop score**			
Mean (SD)	2.30 (1.73)	3.34 (2.40)	0.00
**Induction reason, n (%)**			
Postdate	42 (39.6)	43 (35.2)	0.34
Pre eclampsia	37 (34.9)	35 (28.7)	0.34
Gestational hypertension	9 (8.5)	16 (13.1)	0.32
Other maternal medical conditions	17 (16.0)	23 (18.9)	0.21
Previous unfavourable pregnancy outcome	1 (0.9)	5 (4.1)	-
**Birth weight, kilograms**			
Mean (SD)	2.87 (0.74)	2.88 (0.72)	0.94
**Mode of delivery, n (%)**			
Vaginal deliveries	88 (83.0)	85 (69.7)	0.00
Caesarean section deliveries	18 (17.0)	37 (30.3)	-

With exception of model of delivery and parity, other baseline obstetric and socio-demographic characteristics were similar in the two study groups. Significantly high proportion of women in misoprostol group 88 (83.0%) achieved vaginal delivery-compared to 85 (69.7%) in the dinoprostone group (Table 1).

### 

#### Maternal and Foetal Adverse Outcomes Post Treatment

No differences in maternal and foetal adverse outcomes were observed following labour induction with misoprostol and dinoprostone (See Table 2) Women in both groups experienced good HRQoL outcomes but those in misoprostol group had significantly better HRQoL outcomes than women in dinoprostone group (See Table 3). Women who received misoprostol were more likely to have better HRQoL outcomes than those who received dinoprostone for labour induction. Though some of the outcomes of HRQoL domains did not vary with the type of drug used to induce labour, women in misoprostol group were more likely to experience reduced bodily pain AOR 1.37; 95% CI, 1.07 to 1.71 and physical functioning AOR 4.84; 95% CI, 1.23 to 19.04 (See Table 4).

**TABLE 2.** Maternal and Foetal Adverse Outcomes Post Treatment

Adverse outcomes	Misoprostol (n = 106) n (%)	Dinoprostone (n = 122) n (%)	COR (95% CI)	AOR (95% CI)	P value
Perineal trauma (lacerations, tear)	34 (32.1)	38 (31.1)	0.99 (0.55 – 1.67)	1.54 (0.56 – 4.18)	0.39
Nausea and Vomiting	16 (15.1)	17 (13.9)	0.91 (0.44 – 1.91)		0.80
Diarrhoea	13 (12.3)	23 (18.9)	1.66 (0.79 – 3.47)		0.17
Postpartum haemorrhage	3 (2.8)	1 (0.8)	0.28 (0.29 – 2.77)		0.27
Blood transfusion	5 (4.7)	2 (1.6)	0.34	(0.64 – 1.77)	0.19
Apgar Score <7 at 5 minutes	2 (1.9)	6 (4.9)	2.69 (0.53 – 13.62)	2.29 (0.39 – 13.29)	0.36
Meconium stained liquor	0 (0.0)	3 (2.5)	-	-	-
FHR abnormalities	1 (0.9)	6 (4.9)	1.69 (0.04 – 18.75)	1.70 (0.09 – 18.79)	0.06

COR: Crude Odds Ratio AOR: Adjusted Odds Ratio FHR: Foetal Heart Rate

**TABLE 3.** Mean Scores of Health Related Quality of Life for Each Domain in the Misoprostol And Dinoprostone Study Groups

Domain	Misoprostol (n= 106) Mean (SD)	Dinoprostone (n=122) Mean (SD)	Mean difference	95% CI	P value
Reduced bodily pain	93.76 (9.03)	84.19 (18.33)	9.56	5.70 - 13.42	0.00
Physical functioning	83.64 (15.85)	73.58 (19.38)	10.05	5.39 – 14.72	0.00
Mental health	96.40 (5.00)	93.55 (6.31)	2.84	1.34 – 4.35	0.00
General health	93.78 (9.34)	90.23 (10.69)	3.55	0.92 – 6.19	0.01
Social functioning	94.81 (7.53)	91.25 (8.00)	3.55	1.51 – 5.59	0.00
Satisfaction perceptions	94.96 (10.63)	90.71 (11.43)	4.25	1.36 – 7.15	0.00
Overall HRQoL mean scores	92.89 (6.54)	87.25 (8.83)	5.64	3.58 – 7.69	0.00

**TABLE 4.** Effects of Drug Induced Labour on Health Related Quality of Life Outcomes (Misoprostol compared to Dinoprostone)

Domain	Misoprostol (n = 106) n (%)	Dinoprostone (n =122) n (%)	COR (95% CI)	AOR (95% CI)	P value
Reduced bodily pain	97 (91.5)	85 (69.7)	4.69 (2.14 – 10.28)	1.37 (1.07 – 1.71)	0.00
Physical functioning	71 (67.0)	58 (47.5)	2.24 (1.30 – 3.83)	4.84 (1.23 – 19.04)	0.02
Mental health	104 (98.1)	117 (96.7)	1.77 (0.31 – 9.90)	0.78 (0.73 – 8.30)	0.83
General health	99 (93.4)	108 (88.5)	1.83 (0.71 – 4.72)	1.77 (0.57 – 5.47)	0.31
Social functioning	100 (98.0)	103 (92.0)	3.04 (1.40 – 6.61)	2.92 (0.42 – 20.27)	0.27
Satisfaction perceptions	98 (92.5)	106 (86.9)	1.84 (0.75 – 4.51)	1.44 (0.45 – 4.60)	0.53
Overall HRQoL score	100 (98.0)	97 (80.8)	11.85 (2.72 – 51.64)	10.070 (2.02 – 56.99)	0.01

COR: Crude Odds Ratio AOR: Adjusted Odds Ratio

## DISCUSSION

This study offers important insights into how labour induction with vaginal administration of misoprostol and dinoprostone tablets impact on maternal postpartum HRQoL. It generates hypotheses for the differences in quality of life in the two study groups. Our findings show that women who received misoprostol significantly scored higher in all six domains of HRQoL compared to those who received dinoprostone. These findings indicate that, misoprostol could improve health outcomes of women in the early postpartum period compared to dinoprostone.

Women who received misoprostol scored higher on physical functioning and bodily pain domains compared to those who received dinoprostone. These findings were possibly due to low rate of caesarean deliveries in the misoprostol group. High rates of caesarean deliveries in the dinoprostone group possibly contributed to bodily pain, impaired physical performance and emotional events. Similar findings were observed in previous studies^27,29^, in which women who delivered by emergency caesarean section had worse physical HRQoL scores.

Our study findings also show that women who received misoprostol had higher scores on mental health domain compared to those who received dinoprostone. These findings could be explained by the low incidence of foetal adverse outcomes in the misoprostol group. The proportions of foetal heart rate abnormalities and Apgar score of less than 7 at five minutes were common in the dinoprostone group. Cases of meconium stained liquor occurred only in the dinoprostone group. Worries concerning the wellbeing of the new born perhaps contributed to poor mental HRQoL scores among women in the dinoprostone group.

General health perceptions were measured in terms of feelings of satisfaction or dissatisfaction with one's health status. Misoprostol group had higher general health domain scores compared to dinoprostone group. These findings could be supported by the high scores on reduced bodily pain, physical functioning and mental health domains among women in the misoprostol group. Having enhanced general health, women who received misoprostol were delighted to socially interact and achieve social relations. Comparison with other studies is limited as most studies compare maternal HRQoL between women undergoing induction of labour versus expectant management.

Regarding the mode of delivery, our results show lower incidences of caesarean section deliveries among women in the misoprostol group compared to the dinoprostone group. These findings may be associated to the better HRQoL among women in the misoprostol group reported after adjustment for mode of delivery in the multivariate regression analyses. This is consistent with the natural course of recovery after childbirth. However; these results are inconsistent with a number of previous studies^30,31^. The quality of life domain scores were similar in the vaginal and caesarean section groups at two weeks and six weeks postpartum among primiparas women^30,31^.

Similar findings were reported among multiparas women at eight weeks postpartum^31^. The reason why our results are not consistent with the cited studies may be related to early timing of data collection within twenty four hours following childbirth that we employed in this study.

In this study, women who received misoprostol were highly satisfied with labour induction procedure compared to those who received dinoprostone. These findings were possibly contributed by high rates of vaginal birth and low incidences of maternal–foetal adverse outcomes among the women who received misoprostol. However, satisfaction rates in both study groups were higher compared to the rates reported in previous studies^32,33^. This variation perhaps was due to methodological differences. The cited studies employed a retrospective design while in our study we applied prospective methods. Retrospective design is prone to recall bias. This is more likely to have contributed to the noticeable differences.

There was statistically significant difference in the baseline Bishop Score between the two study groups, with the average score of <3 and >3 in the misoprostol and dinoprostone group respectively. The Bishop Scores of >3 has been associated with increased proportion of vaginal birth in some studies^34,35^. Other studies have reported no difference in the proportion of vaginal birth among patients with Bishop Scores of <3 and >3^36,37^. In this study, low proportion of vaginal birth was observed in a group of patients with Bishop Score of >3. Perhaps the difference in baseline Bishop Scores did not influence the mode of delivery in this study. The major strength of this study is its design. Cross-sectional st udy with data collection within 24 hours of delivery lowered the recall bias, and likely contributed to collection of more accurate information. Power analysis was used to estimate the sample size which may permit generaliz-ability of the results. Our study has a number of limitations. First, due to time limit we assessed HRQoL outcomes within 24 hours post-delivery, although follow up through the puerperium would have generated more findings. Second, we had no women's HRQoL data prior to undergoing labour induction; therefore we have no knowledge of whether women with poor HRQoL after labour induction already presented these levels before or during pregnancy. Therefore longitudinal studies including HRQoL assessment during pregnancy needs to be conducted.

Given the absence of contraindications, the study findings show misoprostol to be the best choice (other factors remaining constant) for induction of labour with enhancement of women's postpartum HRQoL. However, the choice for an optimal labour induction method may also need to be guided by economic evaluation comparison of the interventions. Whether labour induction with misoprostol versus dinoprostone has different cost implications should be part of future research.

## CONCLUSION

In women with clinical indications, labour induction with misoprostol results to better HRQoL post-delivery compared to dinoprostone. Health related quality of life information is of particular value in routine care of natal and postnatal mothers. Current and updated guidelines should address the impacts of labour induction interventions on maternal HRQoL, and encourage the use of quality of life information in provision of holistic natal and postnatal care services. Clinical trials are recommended to determine the effectiveness of labour induction with either of the two methods and address the historical adverse outcomes associated to the use of misoprostol.

## List of Abbreviations

FHR: – Foetal Heart Rate
HRQoL: – Health Related Quality of Life,
MNH: –Muhimbili National Hospital
RCOG: – Royal College of Obstetricians and Gynaecologists
SF-36: – 36-Item Short Form
